# Incidental squamous cell carcinoma of renal pelvis presenting as skin invasion: a case report

**DOI:** 10.1186/s13256-020-02530-6

**Published:** 2020-12-15

**Authors:** Xinghui Sun, Yongqing Li

**Affiliations:** 1 Department of Urology, Affiliated the 900th Hospital of PLA, Fujian Medical University, 156 Xi Er Huan Road, Fuzhou City, Fujian Province 350025 People’s Republic of China; 2 Department of Oncology Radiotherapy, Affiliated the 900th Hospital of PLA, Fujian Medical University, 156 Xi Er Huan Road, Fuzhou City, Fujian Province 350025 People’s Republic of China

**Keywords:** Squamous cell carcinoma, Renal pelvic tumor, Calculus, Renal mass, Skin ulcer

## Abstract

**Introduction:**

Squamous cell carcinoma of the renal pelvis is a rare neoplasm, accounting for less than 0.8% of malignant renal tumors. Chronic irritation is believed to be the primary pathogenic cause for squamous cell carcinoma of the renal pelvis. The most frequently reported cases of squamous cell carcinoma of the renal pelvis generally present with hydronephrosis, pyelonephritis, or nephrolithiasis. The skin of the flank is a very uncommon site of clinical presentation. Here, we report an exceedingly rare case of squamous cell carcinoma of the renal pelvis presenting as skin invasion of the flank.

**Case presentation:**

A 66-year-old Han Chinese man consulted our hospital because of a right lumbar skin lesion lasting more than 3 months. His physical examination revealed that he had a palpable mass about 6.0 cm × 5.0 cm in size at the posterior axillary line in the right low back with skin ulceration 3 mm in diameter and exudation on it. Magnetic resonance imaging showed hydronephrosis of the right kidney and plaque-like abnormal signal in the middle portion of the kidney. The patient underwent a right nephrectomy. The sinus tract formation between the ulcerative skin in the right low back and the middle portion of the right kidney could be found. The distended kidney could not be excised entirely for tight adhesion. Pathological examination showed moderately differentiated renal squamous cell carcinoma with invasion of the renal parenchyma and perirenal adipose tissue.

**Conclusion:**

It is extremely rare for renal squamous cell carcinoma to present as skin invasion. Recurrent percutaneous nephrolithotomy may be a risk factor for squamous cell carcinoma of the renal pelvis. The possibility of renal squamous cell carcinoma should be kept in mind in patients who have hydronephrosis, nephrolithiasis, or chronic pyelonephritis for a long time or with renal anomalies. More radiological examinations are suggested for such patients.

## Introduction

Squamous cell carcinoma (SCC) of the renal pelvis is a rare neoplasm, accounting for less than 0.8% of malignant renal tumors [1]. Chronic irritation leading to squamous metaplasia of urothelium is believed to cause SCC of the renal pelvis [1]. The most frequently reported SCC of the renal pelvis presents with hydronephrosis, pyelonephritis, or nephrolithiasis. The skin of the flank is a very uncommon site of clinical presentation. Here, we report an exceedingly rare case of SCC of the renal pelvis presenting as skin invasion of the flank.

## Case presentation

A 66-year-old Han Chinese man consulted our hospital because of a right lumbar skin lesion with low back pain lasting more than 2 months. He had undergone percutaneous nephrolithotomy (PCNL) in his right kidney 8 years ago and 3 months ago, respectively, and he had a history of diabetes for more than 10 years. No gross hematuria was observed. His physical examination revealed that he had a palpable mass about 6.0 cm × 5.0 cm in size at the posterior axillary line in the right low back with skin ulceration 3 mm in diameter and exudation on it. The patient stated that the ulcerative skin was located at the previous PCNL site.

**Fig. 1:**
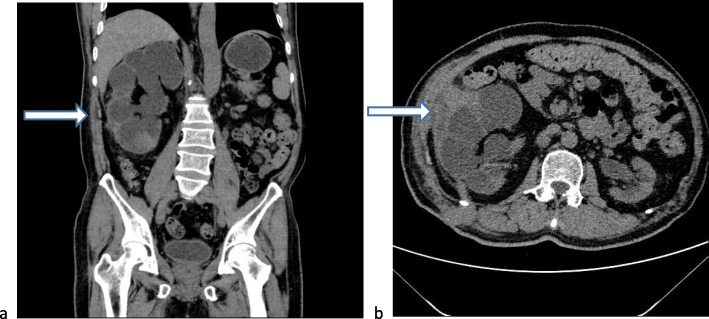
Coronal computed tomography (CT) (**a**) and axial CT (**b**) showing a hydronephrotic right kidney partially adhering to the flank. *White arrow* shows the skin involvement site

**Fig. 2:**
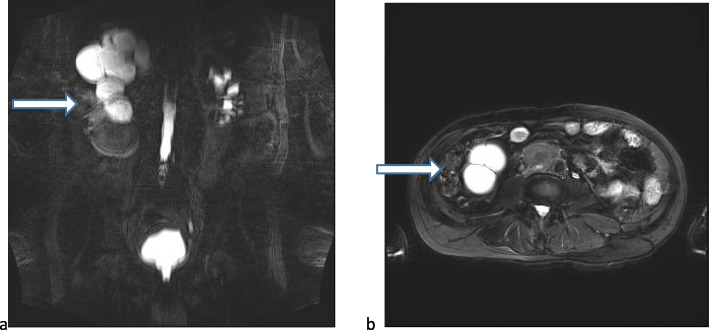
Coronal magnetic resonance imaging (MRI) (**a**) and axial MRI (**b**) showing huge hydronephrosis of the right kidney with plaque-like abnormal signal in the middle portion (malignant mass). *White arrow* shows the mass

Laboratory investigation revealed that the patient’s white blood cell count and serum creatinine level were 11,260/μl and 1.8 mg/dl, respectively. Abdominopelvic computed tomography (CT) revealed right kidney stones with severe hydronephrosis (Fig. 1). Magnetic resonance imaging (MRI) showed hydronephrosis of the right kidney and plaque-like abnormal signal in the middle portion of the right kidney, low signal on T1-weighted imaging, high signal on fat-suppressed T2-weighted imaging and diffusion-weighted imaging, and a significantly decreased apparent diffusion coefficient value. The maximum size of the abnormal signal was 4.8 × 4.3 cm (Fig. 2). Two enlarged lymph nodes with the maximum dimension of 4.8 × 3.9 cm were observed on the right of the aorta in the retroperitoneum. A Tc-99m diethylenetriaminepentaacetic acid renal scan demonstrated that the patient’s estimated glomerular filtration rate was 7.31 ml/min/1.73 in the right kidney and 30.69 ml/min/1.73 in the left, respectively. There was no evidence of distant metastasis. The clinical stage of the patient was considered to be from cT4N1M0 to cT4N2M0.

**Fig. 3:**
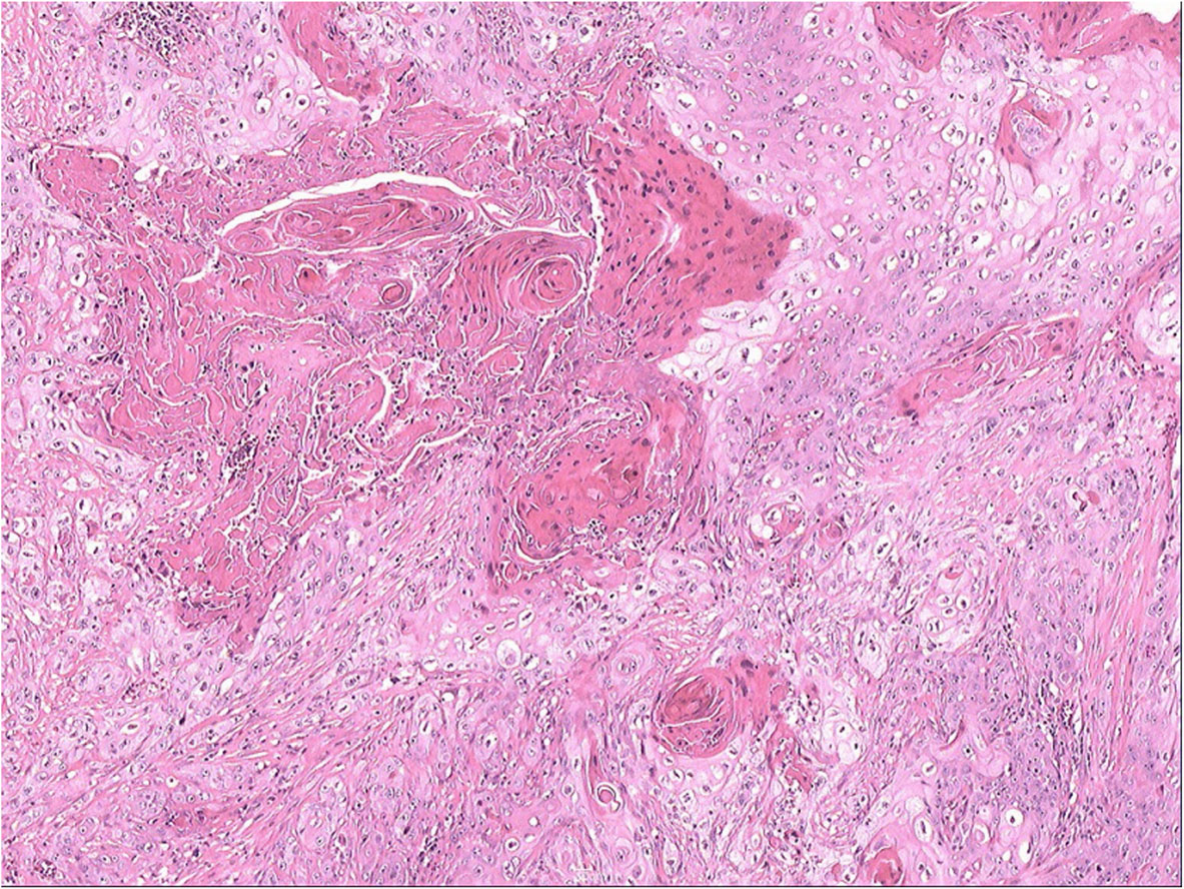
Histopathological examination of the tissue in the area of the right renal pelvis showing well-differentiated to moderately differentiated renal pelvis squamous cell carcinoma (40 x 10 magnifications of the microscope; hematoxylin-eosin staining (H&E) stain)

**Fig. 4:**
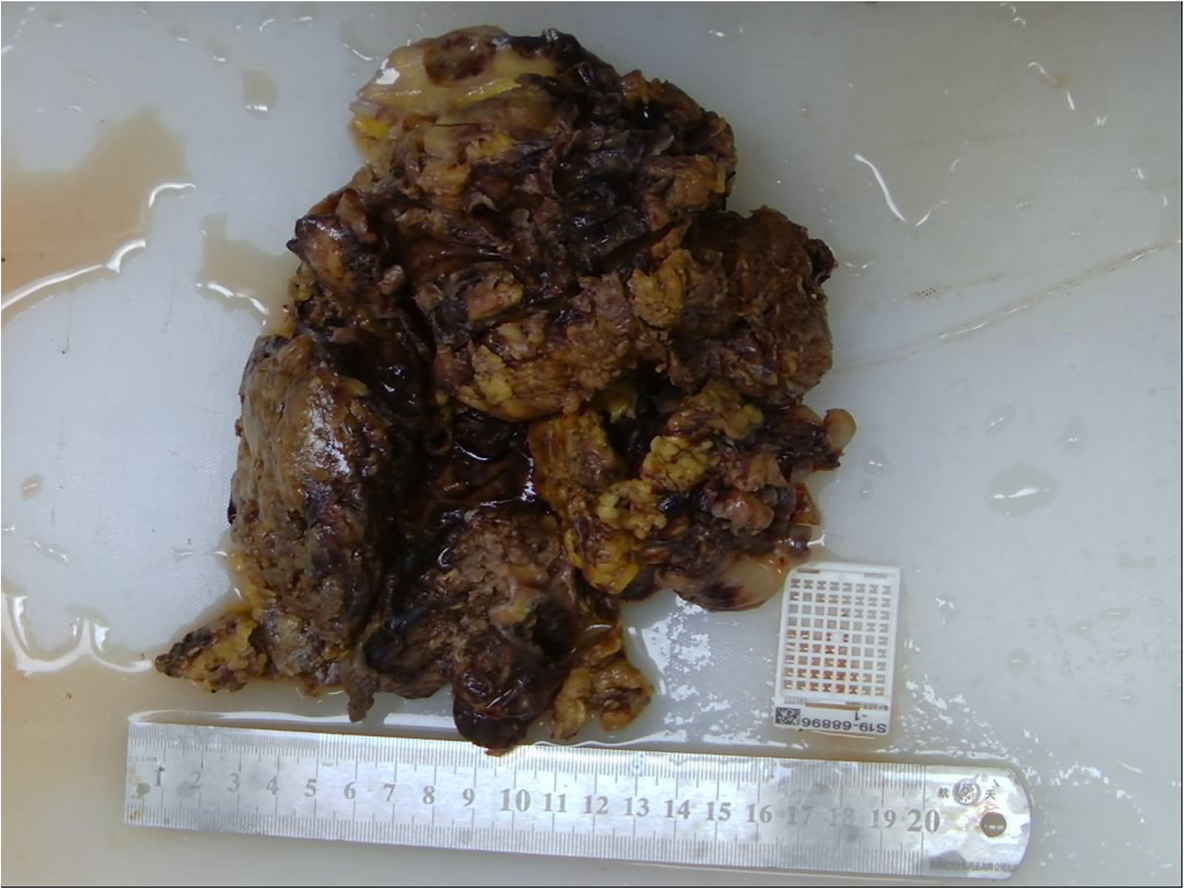
Gross appearance of surgical specimen showing kidney structural destruction

The patient underwent a right open simple nephrectomy under general anesthesia. During surgery, a huge, distended, and saclike kidney measuring 25.0 × 18.0 × 18.0 cm without grossly visible renal tissue was observed to adhere tightly to the adjacent tissue, including the retroperitoneum, psoas muscles, and diaphragm. The sinus tract formation between the ulcerative skin in the right low back and the middle portion of the right kidney could be found. The distended kidney could not be excised entirely for tight adhesion. The lymph nodes adhering to the aorta also could not be resected; only the right upper ureter could be partially resected. The resected specimen was a fragment of renal tissue. Pathological examination revealed moderately differentiated renal SCC with invasion of the renal parenchyma and perirenal adipose tissue (Figs. 3, 4). The patient experienced skin lesion healing and resolution of back pain after surgery. The patient accepted and understood the condition, but he developed local recurrence in the first month of follow-up and was treated with radiotherapy. Radiotherapy was delivered using three-dimensional conformal therapy with a dose of 50 Gy. No adjuvant chemotherapy was given. The patient died 3 months postoperatively.

## Discussion

Primary SCC of the kidney is a rare tumor that commonly originates from the renal pelvis. Chronic pyelonephritis and nephrolithiasis are reported as the two most common etiological factors in renal SCC. Other possible risk factors include schistosomiasis, hydronephrosis, vitamin A deficiency, smoking, and exogenous and endogenous chemicals [2]. It was proposed that chronic irritation of the urothelium mentioned above led to squamous metaplasia and subsequently developed into renal SCC [3–5].

In the early stage, the clinical manifestation of renal SCC is nonspecific. It has been reported that most cases of SCC of the renal pelvis present with hydronephrosis, chronic pyelonephritis, kidney stones, and renal cysts [2, 6–8]. SCC cases also have been discovered in the kidneys with various anomalies such as horseshoe kidney, ectopic kidney, polycystic kidney disease, and renal calyceal diverticulum [2]. It is extremely rare that renal SCC presents as a skin lesion. A fistula between the lumbar skin and the pole of the kidney as presented in this case report has been reported less often. To the best of our knowledge, this is only the third such report in the literature [9, 10]. In our patient, the sinus tract between the previous PCNL site and the superior pole of the right kidney was formed. A similar finding was also observed in other reports [9]. Recurrent PCNL may be a risk factor for squamous metaplasia and subsequent SCC in the kidney with chronic pyelonephritis or nephrolithiasis.

In our patient, initial contrast-enhanced CT was not helpful for the diagnosis of malignant carcinoma. Subsequent MRI led to the diagnosis of a malignant mass. MRI and ^18^F-fluorodeoxyglucose positron emission tomography (PET)/CT may be a useful diagnostic tool to evaluate primary renal pelvic SCC [11]. More radiological examinations are suggested for a suspected renal mass, especially in patients with hydronephrosis, chronic pyelonephritis, stones, and/or renal anomalies. As mentioned above, PET/CT is usually a reliable radiological examination for such soft masses [11]. Percutaneous biopsy also contributes to the diagnosis of renal SCC. There are two previous cases of SCC of the kidney and pelvis diagnosed by fine-needle aspiration cytology reported in the literature [8, 12]. However, the definitive pathology of SCC was only obtained after nephrectomy.

Adjuvant chemotherapy or radiotherapy has no obvious benefits for overall survival of kidney SCC [2]. The rare possibility of renal SCC should be kept in mind for patients who have hydronephrosis, nephrolithiasis, or chronic pyelonephritis for a long time or with renal anomalies. More radiologic examinations are suggested for such patients.

## Conclusion

It is extremely rare that renal SCC presents as skin invasion. The rare possibility of renal SCC should be kept in mind for patients who have hydronephrosis, nephrolithiasis, or chronic pyelonephritis for a long time or with renal anomalies.

## Data Availability

All data generated or analyzed during this study are included in this published article.

## Abbreviations

CT: Computed tomography
MRI: Magnetic resonance imaging
PCNL: Percutaneous nephrolithotomy
SCC: Squamous cell carcinoma

## References

[1] Li MK, Cheung WL. Squamous cell carcinoma of the renal pelvis. J Urol. 1987;138(2):269–71. 10.1016/s0022-5347(17)43116-8 3599235

[2] Jiang P, Wang C, Chen S, Li J, Xiang J, Xie L. Primary renal squamous cell carcinoma mimicking the renal cyst: a case report and review of the recent literature. BMC Urol. 2015;15:69–72. 10.1186/s12894-015-0064-z PMC4511242 26201315

[3] Ogawa M MT, Toyoshima T FM. Squamous cell carcinoma in a duplicated renal pelvis. Int J Clin Exp Pathol. 2014;7(11):7957–61. PMC4270515 25550838

[4] Palmer CJ, Atty C, Sekosan M, Hollowell CM, Wille MA. Squamous cell carcinoma of the renal pelvis. Urology. 2014;84:8–11. 10.1016/j.urology.2013.11.020 24507893

[5] Obad-Kovačević D, Kardum-Skelin I, Kaić G, Jelić-Puškarić B, Kovačević K. Hydronephrotic kidney previously treated for tuberculosis: rare primary squamous cell carcinoma of renal pelvis diagnosed by fine-needle aspiration cytology. Urol Case Rep. 2015;3(5):173–4. 10.1016/j.eucr.2015.06.013 PMC4672648 26793542

[6] Güler Y, Üçpınar B, Erbin A, *et al.* Renal pyelocalyceal squamous cell carcinoma in a patient with an ectopic kidney presenting with chronic pyelonephritis: a case report. J Med Case Rep. 2019;13:154–7. 10.1186/s13256-019-2090-z PMC6532225 31118085

[7] Bandyopadhyay R, Biswas S, Nag D, Ghosh AK. Squamous cell carcinoma of the renal pelvis presenting as hydronephrosis. J Can Res Ther. 2010;6(4):537–9. 10.4103/0973-1482.77060 21358095

[8] Paonessa J, Beck H, Cook S. Squamous cell carcinoma of the renal pelvis associated with kidney stones: a case report. Med Oncol. 2011;28(Suppl 1):S392–4. 10.1007/s12032-010-9704-z 20936378

[9] Kim JR, Jeong YB, Lee NH, Wang SI. Squamous cell carcinoma of the renal pelvis presenting as an integumentary neoplasm of the flank: a case report. Medicine (Baltimore). 2019;98(36):e17049. 10.1097/MD.0000000000017049 PMC6739005 31490399

[10] Slimane M, Hadidane M, Bouzaiene H, Driss M, Jaidane O, Henchiri H, *et al.* Squamous cells carcinoma of the renal pelvis discovered due to parietal and skin invasion: an uncommon manifestation. Pan Afr Med J. 2018;31:246. 10.11604/pamj.2018.31.246.10266 PMC6691301 31448003

[11] Deng S, Zhang B, Huang Y, Li J, Sang S, Zhang W. Case report of primary renal pelvis squamous cell carcinoma coexisting with long-standing calculi in left kidney on ^18^F-FDG PET/CT. Medicine (Baltimore). 2017;96(11):e6341. 10.1097/MD.0000000000006341 PMC5369919 28296764

[12] Bindra R, Gupta S, Gupta N. Cytological diagnosis of squamous cell carcinoma of renal pelvis. J Cytol. 2010;27(2):76–7. 10.4103/0970-9371.70756 PMC3001183 21157557

